# Predicting Drug-Disease Association Based on Ensemble Strategy

**DOI:** 10.3389/fgene.2021.666575

**Published:** 2021-05-03

**Authors:** Jianlin Wang, Wenxiu Wang, Chaokun Yan, Junwei Luo, Ge Zhang

**Affiliations:** ^1^School of Computer and Information Engineering, Henan University, Kaifeng, China; ^2^College of Computer Science and Technology, Henan Polytechnic University, Jiaozuo, China

**Keywords:** drug repositioning, ensemble strategy, similarity measure, matrix completion, drug-disease association

## Abstract

Drug repositioning is used to find new uses for existing drugs, effectively shortening the drug research and development cycle and reducing costs and risks. A new model of drug repositioning based on ensemble learning is proposed. This work develops a novel computational drug repositioning approach called CMAF to discover potential drug-disease associations. First, for new drugs and diseases or unknown drug-disease pairs, based on their known neighbor information, an association probability can be obtained by implementing the weighted K nearest known neighbors (WKNKN) method and improving the drug-disease association information. Then, a new drug similarity network and new disease similarity network can be constructed. Three prediction models are applied and ensembled to enable the final association of drug-disease pairs based on improved drug-disease association information and the constructed similarity network. The experimental results demonstrate that the developed approach outperforms recent state-of-the-art prediction models. Case studies further confirm the predictive ability of the proposed method. Our proposed method can effectively improve the prediction results.

## 1. Introduction

Traditional drug discovery is a high-risk, high-investment, and long-term process (Li et al., 2015). It is well-known that it usually takes more than 10 years and more than $800 million to bring a new drug to market (Adams and Brantner, 2006). Additionally, the probability of drug approval success is below 10% (Ashburn and Thor, 2004). Considering the challenges of traditional drug discovery, the drug repositioning method is rising in popularity (Cano et al., 2017) and has attracted increasing interest from the research community and pharmaceutical industry (Shameer et al., 2015). Some successful repositioning drugs, such as duloxetine, sildenafil, and thalidomide, have generated high revenues in the history of their patent holders or companies (Ashburn and Thor, 2004).

The purpose of drug repositioning is to discover new indications for old drugs. Recently, many computational drug repositioning techniques, such as machine learning-based models, have been used to identify potential drug-disease interactions (Li et al., 2015). For example, Napolitano et al. (2013) melded drug-related features into a single information layer, which was used to train a multi-class support vector machine classifier whose output was a therapeutic class for a given drug. Chen and Li (2017) proposed the flexible and robust multiple-source learning (FRMSL) method to integrate multiple heterogeneous data sources to obtain drug-drug similarity and disease-disease similarity, and used the Kronecker regularized least squares (KronRLS) approach to solve the prediction problem. Liang et al. (2017) used Laplacian regularized sparse subspace learning to find novel drug indications, integrating multiple pieces of information. Most machine learning-based models using negative samples are generated randomly from unknown associations, among which some false negatives may be included, resulting in a biased decision boundary (Liu et al., 2016a).

In recent years, with the rapid advance of high-throughput biology, huge amounts of multi-omic data have been yielded and several databases have been developed to store these valuable data (Chen et al., 2019; Luo et al., 2020). With the development of publicly available drug-related or disease-related databases, the network-based method is widely used in drug repositioning. The network-based method discovered potential drug–disease associations by propagating information in a heterogeneous biological network containing some information about diseases, drugs, or targets (Luo et al., 2018). For example, Yu et al. (2015) used drugs, protein complexes, and diseases to construct a tripartite network, which inferred the association probabilities of drug-disease pairs. Martìnez et al. (2015) developed DrugNet, a model for drug-disease and disease-drug prioritization; a network of interconnected drugs, proteins, and diseases was built, and DrugNet was used for drug repositioning. Luo et al. (2016) utilized drug- and disease-related properties to compute comprehensive similarity measures and the utility bi-random walk (BiRW) algorithm to find new uses for existing drugs. In recent years, the matrix factorization-based method has been successfully applied to biological association prediction, such as lncRNA-disease (Fu et al., 2017; Lan et al., 2020), drug-target (Liu et al., 2016b; Shi et al., 2018), and drug-disease (Zhang et al., 2018). The method can integrate prior information flexibly and integrate much information and many features into the framework to improve the accuracy of prediction. Zhang et al. (2018) developed a similarity-constrained matrix factorization approach (SCMFDD), which utilizes known drug-disease interactions, drug features, and disease features to predict potential drug-disease associations. Gönen and Kaski (2014) developed a new probabilistic method KBMF2MKL, which extended kernelized matrix factorization by incorporating multiple kernel learning. However, association prediction with matrix factorization has some limitations on the accuracy and prediction performance, especially for new diseases or drugs, which are called cold start problems. So, given different prediction approaches, an ensemble method is a promising way to combine their capacity in predicting the associations between drugs and diseases.

In this work, we develop a new drug repositioning model, CMAF, which integrates three methods (matrix factorization-based, label propagation-based, and network consistency projection-based methods) to obtain the final prediction result. To assess the performance of the developed approach, 10-fold cross-validation was implemented, and from the experimental results, we can see that ensemble models can combine different information to achieve high-accuracy performance. The experimental results demonstrate that CMAF obtained better results than the other four recent models in predicting potential drug-disease associations.

## 2. Materials and Methods

**Figure 1:**
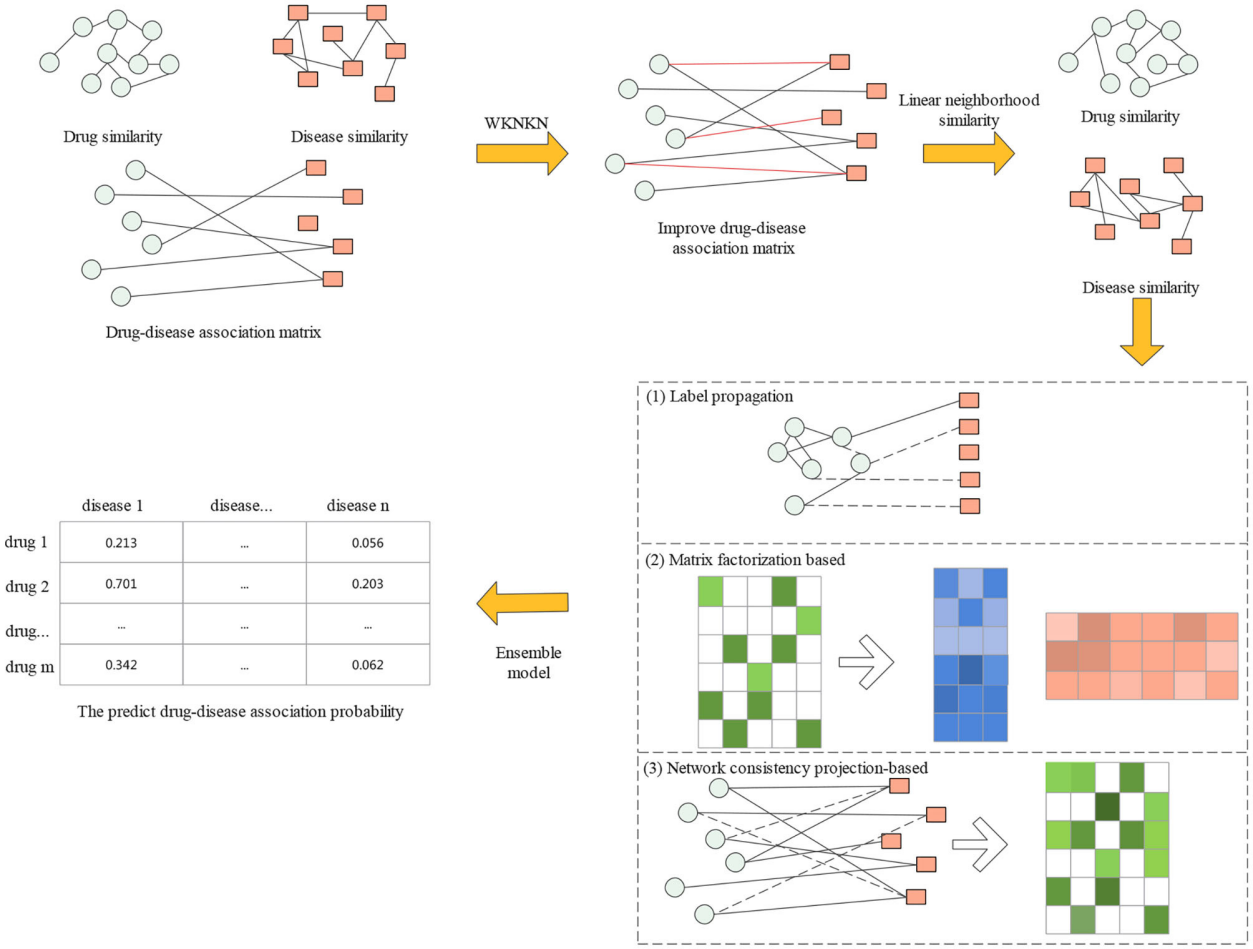
Flowchart of CMAF.

In this section, we first introduce the gold standard dataset used in this study. Then, a proposed drug repositioning method named CMAF is presented to discover new uses for existing drugs. The overall flowchart of CMAF is shown in Figure 1, which contains the following three steps. First, the WKNKN algorithm is used as a preconditioning step to compute the temporary association score for new drugs and diseases or unknown drug-disease pairs. Second, a new drug-drug similarity network and a new disease-disease similarity network can be established. Third, three classical models are used to predict potential drug-disease associations separately, and their prediction results are ensembled to obtain the final association possibility of drug-disease pairs.

### 2.1. Dataset

The dataset used in this paper is curated manually from multiple biological datasets (Gottlieb et al., 2011). The dataset has 593 drugs and 313 diseases involving 1,933 validated drug-disease pairs. The drugs are collected from DrugBank (Wishart et al., 2006), and the diseases are extracted from Online Mendelian Inheritance in Man (OMIM) (Hamosh et al., 2002).

The drug similarity is computed by the Chemical Development Kit (CDK) (Steinbeck et al., 2006) in terms of SMILES (Weininger, 1988) chemical structures, and the similarity between drug pairs is denoted as the Tanimoto score (Tanimoto, 1958) of their 2D chemical fingerprints. The disease similarity is computed using MimMiner (van Driel et al., 2006), which measures the similarity of two diseases by calculating the similarity between the MeSH terms (Lipscomb, 2000) present in the medical description information from the OMIM database.

### 2.2. Improved Drug-disease Association

A known drug-disease association *Y* can be modeled as a two-dimensional matrix, which has *m* drug rows and *n* disease columns, where each entry is denoted by *Y*_*ij*_. The i-th row vector of the adjacency matrix *Y*, *Y*(*r*_*i*_) = (*Y*_*i*1_, *Y*_*i*2_, …, *Y*_*in*_), is the interaction profile for drug *r*_*i*_. Similarly, the j-th column vector of the adjacency matrix *Y*, *Y*(*d*_*j*_) = (*Y*_1*j*_, *Y*_2*j*_, …, *Ymj*), is the interaction profile for disease *d*_*j*_.

It should be noted that the interaction profiles of new drugs or new diseases are all zero values. Additionally, many of the non-associations in *Y* are unobserved situations that could have potential interactions (i.e., false negatives). Therefore, we used WKNKN (Ezzat et al., 2017) to obtain the interaction likelihood value for non-associated drug-disease pairs in terms of their K nearest known neighbors [the K nearest known neighbors can be obtained by the K nearest neighbors (KNN) function according to their drug or disease similarity]. Here, we set K = 5. For every drug *r*_*i*_, the similarity of its chemical structure with the K known drugs nearest to it and their corresponding values in the interaction profiles are utilized to obtain the interaction likelihood profile of the drug *r*_*i*_ as follows:

(1) $$\begin{array}{l} Y_{r}\left(p\right)=\left(\overset{K}{\underset{i=1}{\sum }}w_{i}Y\left(r_{i}\right)\right)/Q_{r} \end{array}$$

where *r*_*i*_ to *r*_*k*_ represent the *K* known nearest neighbors of drug *r*_*p*_; the weight coefficient is $w_{i}=T^{i-1}S^{r}\left(r_{i},r_{p}\right)$ where *T* ≤ 1 is the decay term, and here, we set *T* to 0.5; and $S^{r}\left(r_{i},r_{p}\right)$ is the similarity between *r*_*i*_ and *r*_*p*_. Moreover, $Q_{r}=\overset{K}{\underset{i=1}{\sum }}S^{r}\left(r_{i},r_{p}\right)$ is the normalization term. For the same reason, the interaction likelihood profile of disease *d*_*j*_ is as follows:

(2) $$\begin{array}{l} Y_{d}\left(q\right)=\left(\overset{K}{\underset{j=1}{\sum }}w_{j}Y\left(d_{j}\right)\right)/Q_{d} \end{array}$$

where *d*_1_ to *d*_*k*_ represent the *K* known nearest neighbors of disease *d*_*q*_, the weight coefficient is $w_{j}=T^{j-1}S^{d}\left(d_{j},d_{q}\right)$, the decay term *T* is 0.5, $S^{d}\left(d_{j},d_{q}\right)$ is the similarity between *d*_*j*_ and *d*_*q*_, and the normalization term is $Q_{d}=\overset{K}{\underset{j=1}{\sum }}S^{d}\left(d_{j},d_{q}\right)$.

Then, we fuse *Y*_*r*_ and *Y*_*d*_ to replace *Y*_*ij*_ = 0 by taking the average of the two values mentioned above and denote it as *Y*_*rd*_; we can then obtain a new adjacency matrix *Y*.

(3) $$\begin{array}{l} Y=max\left(Y,Y_{rd}\right) \end{array}$$

where, *Y*_*rd*_ = (*Y*_*r*_ + *Y*_*d*_)/2.

### 2.3. Improved Similarity of Drugs and Diseases

Similarity-based methods are widely used to find similar drugs (Vilar and Hripcsak, 2017). Some studies have shown that the use of similarity measures in drug repositioning often shows high predictive power (Azad et al., 2020). Therefore, similarity measurement is always regarded as an important step in drug repositioning research. The improvement of similarity can improve the prediction performance (Wang and Kurgan, 2019), reduce the computation cost, and make the similarity-based method more attractive and promising (Ding et al., 2014).

Relevant studies found that each data point can be linearly reconstructed from its neighborhood (Wang and Zhang, 2008), we can calculate the pairwise drug similarity and pairwise disease similarity, which is the same method as in previous works (Zhang et al., 2017).

Here, we use drug data points as an example. Let *x*_*i*_ represent the feature vector of the i-th drug. The optimization problem is expressed as:

where *N*(*x*_*i*_) denotes the set of *K*(0 < *K* < *n*) nearest neighbors. Here, we set *K* to 100.

(4) $$\begin{array}{l} \min_{\omega_{i}}\varepsilon_{i}={\left\|x_{i}-\sum_{i_{j}:x_{i}\in N\left(x_{i}\right)}\omega_{i,i_{j}}x_{i_{j}}\right\|}^{2}\\ \;=\sum_{i_{j},i_{k}:x_{i_{j},x_{i_{k}}}\in N\left(x_{i}\right)}\omega_{i,i_{j}}G_{i_{j},i_{k}}^{i}\omega_{i,i_{k}}=\omega_{i}^{T}G^{i}\omega_{i}\\ \text{ s.t. }\sum_{i_{j}:x_{i_{j}}\in N\left(x_{i}\right)}\omega_{i,i_{j}}=1,\omega_{i,i_{j}}\geq 0,j=1,2,\ldots ,K \end{array}$$

$G_{i_{j},i_{l}}^{i}={\left(x_{i}-x_{i_{j}}\right)}^{T}\left(x_{i}-x_{i_{l}}\right)$. *ω*_*i*,_*i*__*j*__ are the weights *x*_*i*_*j*__ for rebuilding *x*_*i*_ and can be seen as the similarity of *x*_*i*_ and *x*_*i*_*j*__.

To avoid over-fitting, we add the regularization term for the rebuilt weight *w*_*i*_ and the objective function can be transformed as follows:

(5) $$\begin{array}{l} \min_{\omega_{i}}\varepsilon_{i}=\omega_{i}^{T}G^{i}\omega_{i}+\text{λ}{\left\|\omega_{i}\right\|}^{2}=\omega_{i}^{T}\left(G^{i}+\text{λ}I\right)\omega_{i}\\ \text{ s.t.}\sum_{i_{j}:x_{i_{j}}\in N\left(x_{i}\right)}\omega_{i,i_{j}}=1,\;\omega_{i,i_{j}}\geq 0,j=1,2,\ldots ,K \end{array}$$

where λ denotes the regularization parameter. Here, we set λ = 1.

We adopt standard quadratic programming to solve Equation (5), and its solution is called the *linear neighborhood similarity*. Here, a weight matrix *W* can be obtained, which we regard as the drug linear neighborhood similarity *S*^r^^*^.

Likewise, we can obtain the disease linear neighborhood similarity *S*^d^^*^.

### 2.4. Prediction Method

In this section, we use the drug linear neighborhood similarity and disease linear neighborhood similarity *S*^d^^*^ to carry out three classical approaches to predict unobserved drug-disease interactions separately and ensemble their prediction results to obtain the final association possibility of drug-disease pairs.

#### 2.4.1. Label Propagation

Label propagation (LP) methods perform the following task: given a weighted network, in which a small part of the nodes are labeled (with labels, such as positive), calculate the labels of the remaining unlabeled nodes (Zhang et al., 2015).

We formulate *S*^d^^*^ as a directed graph, where drugs are nodes and the edge between drug *r*_*i*_ and drug *r*_*j*_ is weighted by the linear neighborhood similarity between the two drugs.

After constructing the graph, we utilize a label propagation approach to predict the unknown drug-disease pair association score (LPRIA). The known drug-disease associations are considered the initial node label information, and then the label information is updated. In each step, each drug node absorbs its neighbor's label information with probability *α* and maintains the initial state with probability 1 − *α*. Here, we set *α* as 0.5. The updated process can be written as:

(6) $$\begin{array}{l} Y_{j}^{t+1}=\alpha S^{r^{*}}Y_{j}^{t}+\left(1-\alpha \right)Y_{j}^{0} \end{array}$$

where, $Y_{j}^{0}$denotes the j-th column of the initial drug-disease interaction matrix *Y* (i.e., the initial states of all drugs for disease *d*_*j*_). Furthermore, taking all diseases into account, the update process can be formulated in matrix form as:

(7) $$\begin{array}{l} Y^{t+1}=\alpha S^{r^{*}}Y^{t}+\left(1-\alpha \right)Y^{0} \end{array}$$

Equation (7) will be used to update the label matrix until it converges, and Equation (7) will converge to:

(8) $$\begin{array}{l} Y^{r^{*}}=\left(1-\alpha \right){\left(I-\alpha S^{r^{*}}\right)}^{-1}Y^{0} \end{array}$$

where I represents the identity matrix and *Y*^r^^*^ represents the predicted drug-disease pair probability from the drug side. For the convergence analysis of this update process, please refer to Wang and Zhang (2008).

Likewise, we constructed the label propagation approach from the disease side to obtain the predicted drug-disease interaction score matrix *Y*^d^^*^. The final association score Y^*^ is obtained according to the average of *Y*^r^^*^ and *Y*^d^^*^.

#### 2.4.2. Non-negative Matrix Factorization

Non-negative matrix factorization (NMF) is an unsupervised model (Fujita et al., 2018). Its goal is to obtain two non-negative matrices and take their product as the optimal approximation to the original matrix. From the perspective of drug repositioning, the drug-disease association matrix *Y* ∈ *R*^*m*×*n*^ is factorized into two non-negative matrices, *W* ∈ *R*^*m*×*k*^ and *H* ∈ *R*^*n*×*k*^ (*k* ≪ min(*m, n*)), here, we set *k* to 100, and *Y* ≈ *WH*^*T*^.

To avoid over-fitting and increase the learning performance, Tikhonov and graph regularization terms are added to the standard NMF model to predict novel drug-disease pairs (NMFRIA). NMFRIA's objective function is as follows:

(9) $$\begin{array}{l} \min_{W,H}{\left\|Y-WH^{T}\right\|}_{F}^{2}+\lambda_{l}\left(\|W{\|}_{F}^{2}+\|H{\|}_{F}^{2}\right)+\lambda_{r}\mathrm{Tr}\left(W^{T}L_{r}W\right)\\ \;+\lambda_{d}\mathrm{Tr}\left(H^{T}L_{d}H\right)\\ \text{ s.t. }W\geq 0,H\geq 0 \end{array}$$

where λ_*l*_, λ_*r*_, and λ_*d*_ represent the regularization coefficients; *Tr*(·) denotes the trace of a matrix, $L_{r}=D_{r}-S^{r^{*}}$ is the graph Laplacian matrix for the drug similarity matrices, *S*^r^^*^ and $L_{d}=D_{d}-S^{d^{*}}$ are the graph Laplacian matrices for the disease similarity matrices *S*^d^^*^ (Liu et al., 2014); and *D*_*r*_ and *D*_*d*_ represent the diagonal matrices whose entries are the row sums of *S*^r^^*^ and *S*^d^^*^, respectively.

The method proposed by Xiao et al. (2018) is adopted to solve the minimization problem, and *W* and *H* are updated with an iterative equation. Here, the updating rules can be defined as:

(10) $$\begin{array}{l} w_{ik}\leftarrow w_{ik}\frac{{\left(YH+\lambda_{r}S^{r^{*}}W\right)}_{ik}}{{\left(WH^{T}H+\lambda_{l}W+\lambda_{r}D_{r}W\right)}_{ik}} \end{array}$$

(11) $$\begin{array}{l} h_{jk}\leftarrow h_{jk}\frac{{\left(Y^{T}W+\lambda_{d}S^{d^{*}}H\right)}_{jk}}{{\left(HW^{T}W+\lambda_{l}H+\lambda_{d}D_{d}H\right)}_{jk}} \end{array}$$

where *w*_*ik*_ represents the i-th row and the k-th column of non-negative matrix *W*, and *h*_*jk*_ represents the j-th row and the k-th column of non-negative matrix *H*.

According to Equations (10) and (11) the two non-negative matrices *W* and *H* are updated until convergence, and then we can obtain the predicted drug-disease interaction matrix as Y^**^ = *WH*^*T*^. Here, we set λ_*l*_ to 2, and λ_*r*_ = λ_*d*_ = 0.0001.

#### 2.4.3. Network Consistency Projection

Network consistency projection (NCP) considers drugs *r*_*i*_ that have a higher similarity to other drugs in the drug similarity matrix; the more drugs are associated with disease *d*_*j*_, the higher the spatial similarity of drug *r*_*i*_ with disease *d*_*j*_ (and vice versa). Here, we use the NCP approach (Gu et al., 2016) for drug-disease association (NCPRIA) to obtain the predicted association scores between unknown drug-disease pairs.

NCPRIA computes the association probability between drug *r*_*i*_ and disease *d*_*j*_ by fusing two network consistency projection scores (the drug and disease space projection scores). Considering that unknown drug-disease pairs are not confirmed by experiment, which cannot prove that they are unrelated, and to prevent 0 from being the denominator, we replace 0 in the matrix *Y* with 10–30.

The drug space projection is the projection of the drug similarity network *S*^r^^*^ on the drug-disease interaction network *Y*, which can be described as follows:

(12) $$\begin{array}{l} NCP_{-}R\left(i,j\right)=\frac{S^{r^{*}}{\left(i,:\right)}^{*}Y\left(:,j\right)}{\left|Y\left(:,j\right)\right|} \end{array}$$

where *S*^r^^*^(*i*, :) denotes the similarities between drug *r*_*i*_ and all other drugs in the i-th row of matrix *S*^r^^*^ and *Y*(:, *j*) denotes the associations between disease *d*_*j*_ and all drugs. |*Y*(:, *j*)| represents the length of the vector *Y*(:, *j*). *NCP*_*R*(*i, j*) represents the network consistency projection score of *S*^r^^*^(*i*, :) on *Y*(:, *j*). It is worth noting that the smaller the angle is between *S*^r^^*^(*i*, :) and *Y*(:, *j*), the more drugs are related to disease *j* and the more similar drugs there are to drug *i*, the larger the network consistency projection score *NCP*_*R*(*i, j*).

Similarly, we can obtain the disease space projection score as follows:

(13) $$\begin{array}{l} NCP_{-}D\left(i,j\right)=\frac{Y{\left(i,:\right)}^{*}S^{d^{*}}\left(:,j\right)}{\left|Y\left(i,:\right)\right|} \end{array}$$

where *S*^d^^*^(:, *j*) denotes the j-th column of matrix *S*^d^^*^ and *Y*(*i*, :) denotes the i-th row of drug-disease association *Y*. *NCP*_*D*(*i, j*) represents the network consistency projection score of *S*^d^^*^(:, *j*) on *Y*(*i*, :).

Finally, the projection score for the drug space and disease space are fused and normalized as follows:

(14) $$\begin{array}{l} Y^{***}\left(i,j\right)=\frac{NCP_{-}R\left(i,j\right)+NCP_{-}D\left(i,j\right)}{\left|S^{r^{*}}\left(i,:\right)|+|S^{d^{*}}\left(:,j\right)\right|} \end{array}$$

where Y^***^ represents the predicted drug-disease association matrix and Y^***^(*i, j*) is the final predicted score of drug *r*_*i*_ and disease *d*_*j*_.

#### 2.4.4. Integrating the Prediction Results

According to the three aforementioned computational drug repositioning methods, to obtain better performance, a fusion model is adopted to integrate their predicted results, and the final prediction score between drugs and diseases is computed as follows:

(15) $$\begin{array}{l} Rt=1-\left(1-Y^{*}\right)\left(1-Y^{**}\right)\left(1-Y^{***}\right) \end{array}$$

In particular, Y^*^ is the predicted drug-disease association probability of the LPRIA method, Y^**^ is the predicted association probability of the NMFRIA method, Y^***^ is the predicted association probability of the NCPRIA method, and *Rt* stands for the final predicted drug-disease association probability.

## 3. Experiments and Results

In this section, the performance of our approach, CMAF, is systematically evaluated. First, we describe the evaluation metrics. Based on a gold standard dataset, we compare our approach with several recent prediction algorithms and present the results in this section. In addition, the effectiveness of the developed method is further confirmed by case studies.

### 3.1. Evaluation Metrics

To evaluate the prediction performance of the proposed CMAF method, 10-fold cross-validation was conducted on the gold standard dataset. In each round of 10-fold cross-validation, all the recorded drug-disease pairs were randomly divided into 10 equal-sized parts. Each part was taken as a test set in turn, while the remaining nine parts of the data were merged as the training set, thus generating 10 pairs of training sets and test sets. To obtain convincing results, 10-fold cross-validation was repeated 10 times, and the average value of 10-folds was taken as the final result. After performing association prediction based on the training set, we can obtain the prediction values for each association. Then, for each drug, the test drug-disease associations are ranked together with all unconfirmed drug-disease pairs (candidate associations) in descending order according to the predicted values. For each specific ranking threshold, four metrics: true positive (TP), false negative (FN), false positive (FP), and true negative (TN), can be obtained based on the ranking results. If a test association has a higher rank value than the given threshold, it is considered as a correctly identified positive sample. Likewise, a candidate association is considered a correctly identified negative sample if it has a lower rank than the given threshold.

To provide an intuitive explanation of the evaluation metrics, a confusion matrix is first defined, which is built by comparing actual values with predicted outcomes. The two classes are constructed with positives and negatives, as shown in Table 1.

**Table 1:** Confusion matrix.

		**Actual value**	
		**Positive**	**Negative**
Predicted value	Positive	True positive (TP)	False negative (FN)
	Negative	False positive (FP)	True negative (TN)

Next, the evaluation metrics of the true positive rate (TPR) and false positive rate (FPR) can be defined as follows:

(16) $$\begin{array}{l} TPR=\frac{TP}{TP+FN} \end{array}$$

(17) $$\begin{array}{l} FPR=\frac{FP}{FP+TN} \end{array}$$

Where TP and FP represent the numbers of correctly and wrongly identified positive samples and TN and FN represent the numbers of correctly and wrongly identified negative samples; TPR and FPR are calculated based on these four metrics. Furthermore, TPR is the ratio of known drug-disease pairs that are correctly predicted, and FPR is the proportion of unconfirmed drug-disease pairs that are predicted.

After that, the receiver operating characteristic (ROC) curve can be drawn based on TPR and FPR at different thresholds. Meanwhile, the area under ROC (AUC) can be calculated to evaluate the prediction performance. The larger the value of the AUC, the better the prediction performance. For instance, if the value of the AUC is equal to 1, it means the best performance.

### 3.2. Comparison With Other Methods

In this section, to evaluate the ability of the proposed approach, we compare CMAF with four other recently proposed computational drug repositioning approaches: NBI (Cheng et al., 2012), BNNR (Yang et al., 2019), HGBI (Wang et al., 2013), and NGRHMDA (Huang et al., 2017). NBI is based on a bipartite network and constructs a two-step diffusion model for drug repositioning (Cheng et al., 2012). BNNR was developed to utilize a bounded nuclear norm regularization approach to construct the drug-disease matrix under the low-rank assumption (Yang et al., 2019). HGBI was proposed according to the guilt-by-association principle and an intuitive interpretation of information flow on a heterogeneous graph (Wang et al., 2013). NGRHMDA uses neighbor-based collaborative filtering and a graph-based scoring method to obtain the association score (Huang et al., 2017). Although HGBI and NBI were originally used to predict potential drug-target associations and NGRHMDA was originally used to predict new microbe-disease associations, they can also be used to predict new drug-disease associations. The parameter values used in NBI, BNNR, HGBI, and NGRHMDA are set based on their corresponding literature.

**Figure 2:**
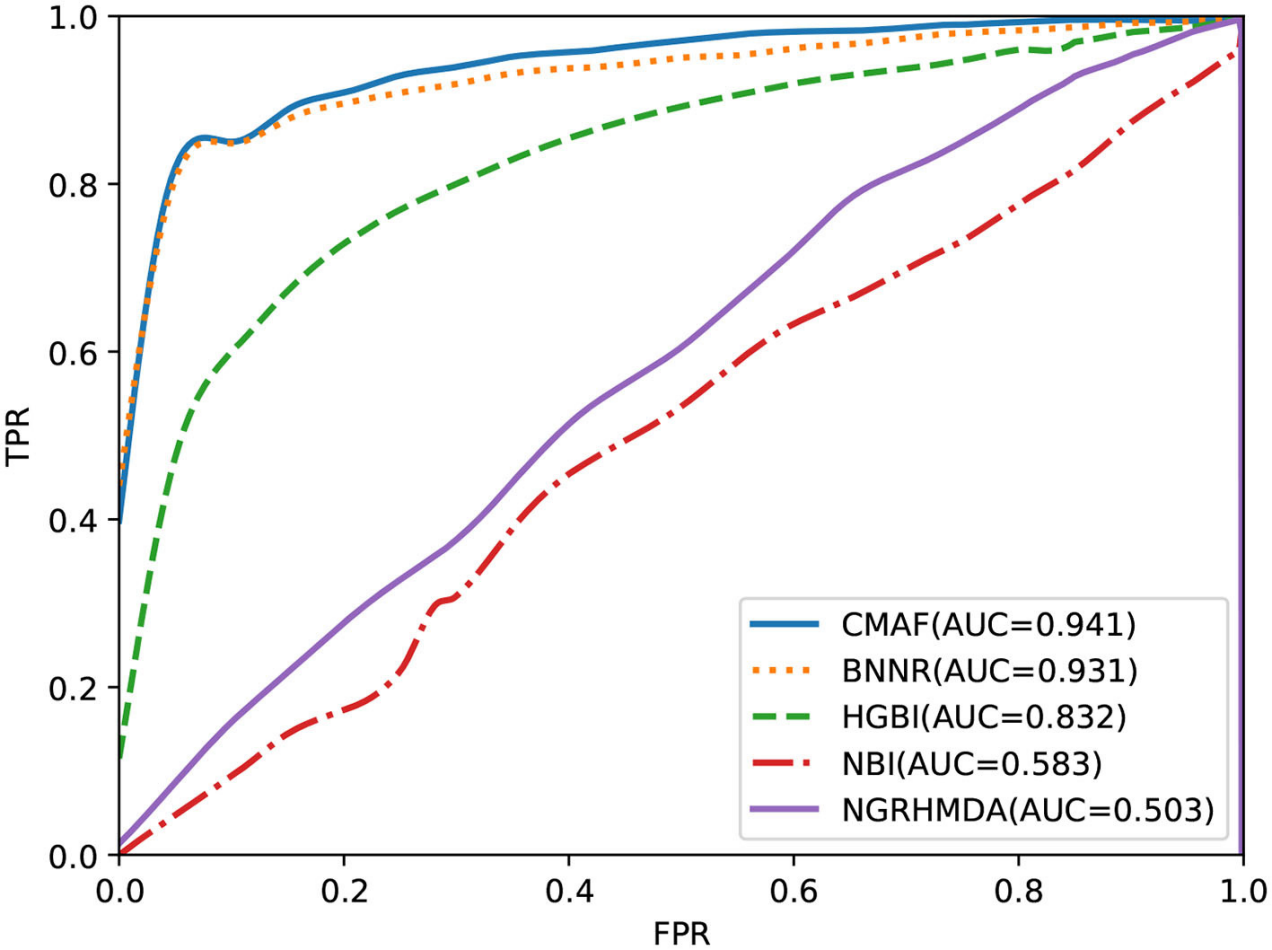
Prediction results of various methods according to ROC curve analysis.

The predictive ability of all drug repositioning approaches is evaluated in terms of the AUC specified in section 3.1. As shown in Figure 2, the results demonstrate that our developed approach, CMAF, is superior to the other four drug repositioning approaches. In detail, CMAF obtains an AUC value of 0.941, while BNNR, HGBI, NBI, and NGRHMDA achieve inferior results of 0.931, 0.832, 0.583, and 0.503, respectively.

### 3.3. Comparison of the Three Methods With Their Combined Model

**Figure 3:**
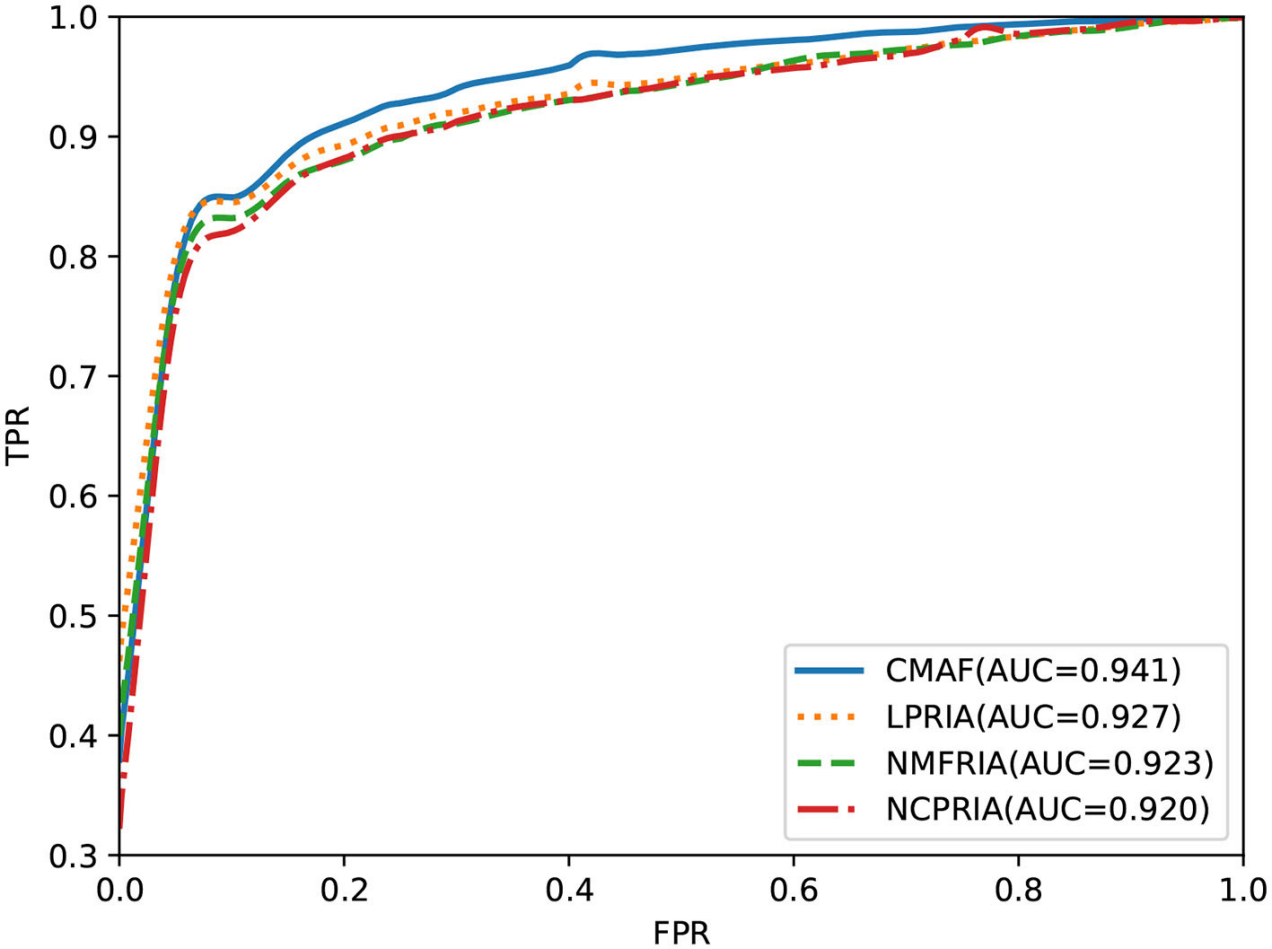
Prediction performance of CMAF and the three individual methods according to the ROC curve.

The effectiveness of the fusion method is evaluated in this section. We performed drug-disease association prediction on the gold standard dataset by using three methods (i.e., the LPRIA, NMFRIA, and NCPRIA methods) and their combined method. As shown in Figure 3, the AUC values of the three methods LPRIA, NMFRIA, and NCPRIA were 0.927, 0.923, and 0.920, respectively; however, the fusion method CMAF obtained an AUC value of 0.941. The experimental results illustrated the effectiveness of our fusion approach. Specifically, the CMAF method obtained the best performance among these four methods.

### 3.4. Prediction for New Drugs

**Figure 4:**
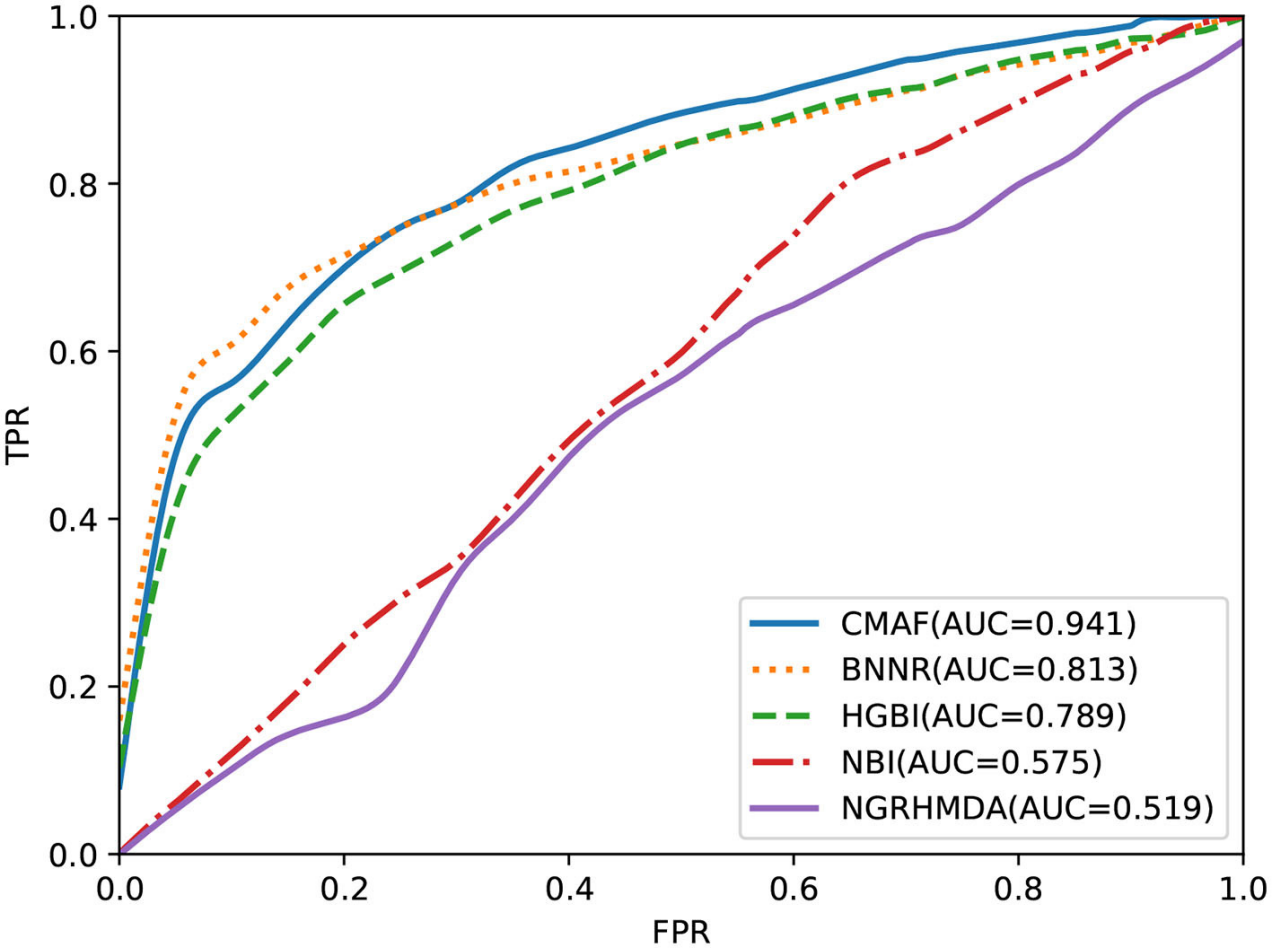
Prediction performance of CMAF and the other four methods in predicting drug-disease associations for new drugs according to the ROC curves.

To test the predictive performance of CMAF for new drugs, a de novo prediction test was executed. In de novo drug validation, for each of the drugs, we deleted all of its known associations, and they were used for testing samples in turn; the other known drug-disease association was used as the training sample. The rankings of the removed drug-disease associations relative to the drug candidate associations were obtained by *de novo* testing, which was used to assess the predictive performance. To compare the predictive ability of different methods in *de novo* testing of new drugs, the other four prediction methods also underwent de novo prediction tests. The experimental results are shown in Figure 4, and the graph demonstrates that our CMAF is superior to the other approaches. In detail, CMAF obtains an AUC value of 0.941, while the results of BNNR, HGBI, NBI, and NGRHMDA are 0.813, 0.789, 0.575, and 0.519, respectively.

## 4. Case Studies

After verifying the predicted performance of CMAF in terms of 10-fold cross-validation, the ability of our proposed model to identify new indications for a given drug is further validated here. To predict new drug-disease interactions, all known drug-disease pairs are considered as the training set, and the remaining unknown drug-disease pairs form the candidate set. By applying our CMAF method, we can obtain all the candidates' set prediction scores. According to the prediction scores, for every drug, all the candidate diseases are ranked.

As an example, we selected some drugs and the corresponding top five candidate diseases as verified information, and then we found that some of them were confirmed in the KEGG (Kanehisa et al., 2013), DrugBank and CTD (Davis et al., 2014) databases, as shown in Table 2. For example, the effectiveness of levodopa in treating Parkinson's disease (PD) due to its ability to cross the blood-brain barrier can be retrieved from the KEGG, DrugBank, and CTD databases. In addition, relevant literature has shown that levodopa-treated patients have gained improvement in most Parkinsonian features in the past half-century (Lewitt, 2015). Flecainide is helpful for treating atrial fibrillation, as can be retrieved from CTD, and there is literature to prove that in clinical trials and real-world use, flecainide is more effective than other antiarrhythmic drugs (AADs) for the acute termination of recent-onset atrial fibrillation (Echt and Ruskin, 2020). From KEGG and CTD, zoledronic acid can be found to treat and prevent multiple forms of osteoporosis. There is also literature to prove that zoledronic acid administered once yearly for up to 3 years improved bone mineral density (BMD) at several skeletal sites, reduced fracture risk and bone turnover, and/or preserved bone structure and mass relative to placebo in clinical studies in patients with primary or secondary osteoporosis (Dhillon, 2016). Amantadine is an antiviral that can be used to cure PD and can be retrieved from KEGG, DB, and CTD. Relevant literature suggests that amantadine is an old antiviral compound that moderately ameliorates impaired motor behavior in Parkinson's disease (Müller et al., 2019).

**Table 2:** Case studies of four chosen drugs: levodopa, flecainide, zoledronic acid, and amantadine.

**Drug (DrugBank IDs)**	**Top 5 candidate diseases (OMIM IDs)**	**Evidence**
DB01235	168600	KEGG/DB/CTD
Levodopa	125320	DB/CTD
	165199	
	254770	
	190400	
DB01195	608583	CTD
Flecainide	194200	KEGG/CTD
	115000	DB/CTD
	157300	
	608622	CTD
DB00399	166710	KEGG/CTD
Zoledronic acid	102400	
	144700	CTD
	166300	
	114480	CTD
DB00915	168600	KEGG/DB/CTD
Amantadine	125320	DB/CTD
	605055	
	104300	CTD
	607225	

*For each drug, the top five ranked predicted drugs are listed below*.

## 5. Conclusion

This work proposed a new computational drug repositioning model named CMAF to find new uses for existing drugs. First, the number of known drug-disease interactions is far less than that of unknown drug-disease interactions in practice, which leads to the problem of data sparseness for drug repositioning. Therefore, we used the WKNKN method as a pre-processing step to compute the temporary association scores for these unknown drug-disease interactions in terms of their known neighbors, and then we computed the linear neighborhood similarity for drugs and diseases. After that, the LPRIA, NMFRIA, and NCPRIA methods were adopted to obtain three predictive association possibilities. Finally, we adopted an ensemble strategy to fuse these three prediction models to obtain the hopefully final prediction result. Compared with several recent computational drug repositioning models, our proposed CMAF approach achieves better predictive performance.

Even though our proposed method obtains promising results, it still has some limitations. First, we plan to consider integrating more predictive methods into the ensemble strategy. Second, CMAF utilizes only single drug-drug similarity and disease-disease similarity to construct prediction methods. In the future, we will compute multiple drug-drug similarities and disease-disease similarities and combine diverse similarities to further improve the predictive performance.

## Data Availability Statement

The original contributions presented in the study are included in the article/supplementary material, further inquiries can be directed to the corresponding author/s.

## Author Contributions

CY and JW conceived and designed the approach. WW performed the experiments. JL analyzed the data. GZ and WW wrote the manuscript. CY and GZ supervised the whole study process and revised the manuscript. All authors have read and approved the final version of manuscript.

## Conflict of Interest

The authors declare that the research was conducted in the absence of any commercial or financial relationships that could be construed as a potential conflict of interest.
